# *Peumus boldus* Extract Inhibits Lipid Accumulation in 3T3-L1 Adipocytes

**DOI:** 10.3390/ijms26094326

**Published:** 2025-05-02

**Authors:** Laura Montaldo, Llerson Bendezu Meza, Mauricio De Marzi, Liliana Noemi Guerra

**Affiliations:** 1 Departamento de Ciencias Basicas, Universidad Nacional de Lujan, Av Constitucion y Ruta 5, Lujan 6700, Buenos Aires, Argentina; laura.montaldo24@gmail.com (L.M.); antti-mage_96@hotmail.com (L.B.M.); mauriciocesardemarzi@gmail.com (M.D.M.); 2 CONICET-INEDES, Grupo de Investigaciones Basicas y Aplicadas en Inmunologıa y Bioactivos (GIBAIB), Av Constitucion y Ruta 5, Lujan 6700, Buenos Aires, Argentina

**Keywords:** antioxidants, polyphenols, oxidative stress, adipocytes

## Abstract

Obesity is a metabolic condition of epidemic scale. Previously, we showed that antioxidant extracts from *Ribes nigrum* had antioxidant and anti-adipogenic effects in mature adipocytes (AD). Here, we evaluated an aqueous extract from *Peumus boldus* (Boldo) in AD and studied its effect on reactive oxygen species (ROS) and lipid production. We analyzed the antioxidant activity (AA) of the Boldo extract using the DPPH technique and polyphenol (Pph) content via Folin’s reagent. In AD, we evaluated ROS production, catalase (CAT) activity, intracellular triglyceride (Tg) and cholesterol (Chol) contents, nitric oxide (NO) production via Griess reagent, and the levels of glycerol (Gly) and TNF-α released in the culture medium. We showed that the Boldo extract has high AA. In vitro, Boldo treatment decreased ROS intracellular production and CAT activity. In addition, the Boldo extract was effective in reducing Tg and Chol levels and NO production. We did not identify significant differences in Gly released or TNF-α secreted. We suggest that the Boldo extract has antioxidant and anti-adipogenic effects, but we did not observe lipolytic effects. Boldo did not modify inflammatory markers.

## 1. Introduction

Obesity is typically defined using the Body Mass Index (BMI). This metric is determined by taking a person’s weight in kilograms and dividing it by the square of their height in meters (kg/m^2^) [1]. According to the World Health Organization (WHO), in 2022, one in eight individuals in the world were affected by obesity, while 43% of adults aged 18 years and over were overweight and 16% were living with obesity. More than 390 million children and adolescents aged 5 to 19 were classified as overweight, with 160 million of them living with obesity (https://www.who.int/news-room/fact-sheets/detail/obesity-and-overweight (accessed on 1 March 2024)). The food habits and physical activity of individuals are highly influenced by environmental and societal factors that limit personal choices. Obesity should be viewed as a collective rather than an individual problem, with the WHO recognizing the urgent need to address the global obesity crisis.

The 3T3-L1 preadipocyte cell line was originally established through clonal expansion from 3T3 cells derived from Swiss mice [2]. Given its ability to differentiate from fibroblast-like cells into adipocytes, the 3T3-L1 cell line has been a good model for studying adipogenesis and fat cell development [3]. This cell line has the potential to differentiate into mature adipocytes in response to dexamethasone, insulin, and 3-isobutyl-1-methylxanthine. A majority of cells show an increase in lipid droplets and intracellular triglyceride levels seven days after initiating the differentiation procedure [4].

Bioactive compounds are produced by plants with different chemical structures capable of modulating varied molecular targets and employed as treatments for various diseases [5]. Polyphenols (Pphs) are a group of bioactive compounds found in vegetables, fruits, beverages, and grains. These natural compounds can be further classified into flavonoids, phenolic acids, among others [6]. Phenolic acids have shown significantly greater in vitro antioxidant activity compared to well-known antioxidant vitamins [7]. Syringic acid (SA; 3,5-dimethoxy-4-benzoic acid) is a phenolic acid derived from benzoic acid that inhibits the clonal expansion of preadipocytes and adipogenesis and shows strong in vitro antioxidant properties [8]. Quercitrin (QU), isoquercitrin (IQ), and afzelin (AF) are flavonoids obtained from *Acer okamotoanum,* which have been shown to possess several biological activities, including antioxidant properties. QU, IQ, and AF effectively decreased Tg intracellular content in 3T3-L1 adipocytes, suggesting their promising potential as anti-obesity agents through the inhibition of lipid accumulation [9].

In the last few decades, medicinal plants have been studied to identify new bioactive compounds that could lead to effective drugs. *Ilex paraguariensis*, known popularly as Yerba Mate (YM) in South America, has been extensively studied. It has high contents of polyphenols (chlorogenic and caffeic acids) and flavonoids (quercetin and rutin) and exhibits antioxidant activity. YM crude extract and its bioactive compounds reduced lipid storage in adipocytes, partly by downregulating the expression of several genes associated with adipogenesis, such as PPAR-γ, CBP-β, and Leptin [10]. Studying plant species in our region is important for this type of plant.

*Peumus boldus*, usually known as Boldo, is an endemic plant from Chile. It has been utilized in traditional medicine for its various biological effects, including anti-inflammatory, hepatoprotective, and antioxidant properties. Its biological effects could be attributed to its powerful ability to scavenge free radicals [11]. Phenolic compounds (polyphenols and flavonoids) are the principal bioactive present in aqueous extracts of *P. boldus* [12]; other components such as the alkaloid boldine have not been detected due to their volatile properties [13]. Few reports described the effects of Boldo aqueous extracts on cellular redox metabolism: one showed a decrease on lipid peroxidation in mice liver during cisplatin treatment [14], while Boldo extracts effects on the molecular mechanism of adipocyte differentiation remain to be elucidated.

Several studies have shown that during the differentiation of 3T3-L1 adipocytes, there is an increase in reactive oxygen species (ROS) production. The redox condition influences mitotic clonal expansion, which is necessary in the early phases of the differentiation process. 3T3-L1 cells stimulated with hormones and hydrogen peroxide (H_2_O_2_) accelerate the progression of the cell cycle. This suggests that ROS production plays a crucial role in the differentiation of preadipocytes [15]. The exposition of 3T3-L1 preadipocytes to H_2_O_2_ halted proliferation, which was related to an increase in mitochondrial biogenesis. Treating cells with polyphenols or flavonoids caused mitochondrial changes, which had a protective effect against oxidative damage [16]. The effects observed under pretreatment conditions could be attributed to the interaction of polyphenols with cell membranes. In addition to the free radical scavenging activity of these compounds, other mechanisms have been suggested to explain their biological effects, including their interactions with membrane lipids and proteins. Furthermore, scavenging ROS has been found to inhibit this process [17].

Nitric oxide (NO) is produced through the oxidation of L-arginine by various isoforms of nitric oxide synthase (NOS). It serves as a key signaling molecule that regulates a wide range of cellular functions [18]. The expression of adipocyte-specific transcription factors such as Peroxisome Proliferator-Activated Receptor γ (PPARγ) maintains the mature adipocyte state. PPARγ is essential for adipocyte differentiation, as it binds to a specific site in the promoter of the terminal differentiation protein aP2 gene, leading to its expression in adipocytes [4]. In addition, PPARγ regulates lipoprotein lipase (LPL). PPARg directly stimulates the expressions of aP2 and LPL genes while exogenous NO decreases the expression of aP2 and LPL mRNA, thus suggesting that NO suppresses adipocyte differentiation by inhibiting the function of PPARg [19].

Tumor necrosis factor-alpha (TNF-α) is a versatile cytokine that plays a key role in regulating various cellular and biological processes, including immune function, cell differentiation, proliferation, apoptosis, and energy metabolism. In the case of obesity, it is known that TNF-α increases because of the chronic inflammation developed during this pathology [20]. Mature adipocytes and macrophages secrete TNF-α in adipose tissue, and free fatty acids (FFAs) such as palmitic acid could be responsible for the increase above the basal level in 3T3-L1 adipocytes [21]. Antioxidant treatment with Ginsenoside bioactive was effective in decreasing TNF-α in FFA-treated 3T3-L1 adipocytes [22,23]. Moreover, LPS could activate the TNF-α secretion pathway [24].

Phenolic compounds have the potential to decrease, prevent, and repair damage caused by oxidative stress and the inflammation associated with metabolic syndrome. Numerous studies have related a higher intake of fruits and vegetables rich in antioxidant molecules with a decrease in chronic diseases, such as diabetes and cardiovascular conditions [23]. Polyphenol-rich extracts derived from chokeberries, raspberries, bilberries, and cranberries could help to prevent or treat obesity by inhibiting adipogenesis, reducing lipid accumulation, and decreasing ROS production in adipocytes [25].

Previously, we investigated an enzymatic extract obtained from *Ribes nigrum* (Cassis). Cassis extract (EC) showed high antioxidant activity and elevated polyphenol and flavonoid contents. We evaluated its anti-adipogenic effect and proved that EC decreased triglyceride and cholesterol intracellular contents compared to non-treated adipocytes [26]. Considering this approach, here we performed studies with an aqueous extract from an important plant commonly consumed in our region, *Peumus boldus*, which has some compounds in common with *Ribes nigrum,* such us polyphenols and flavonoids. Since these vegetable species are rich in bioactive compounds with antioxidant capacities, our aim was to evaluate the in vitro anti-adipogenic effects of *Peumus boldus*.

## 2. Results

### 2.1. Preparation and Characterization of EXTs

We prepared Boldo (BOL) and Quebracho (QUE) extracts. Different properties of the samples were evaluated. Acidity, antioxidant activity, polyphenol, and flavonoid content were determined in samples obtained from the original extract (0.01 g of each plant material incubated 1 mL of PBS for 1 h at 37 °C).

#### 2.1.1. Acidity Measurements

The pH values of EXTs were similar in the samples: 6.27 (BOL) and 6.05 (QUE).

#### 2.1.2. Antioxidant Activity and Polyphenol and Flavonoid Content

It has been reported that antioxidants are beneficial to human health. Since the antioxidant activity of polyphenols is very well known, we considered it important to determine the antiradical capacity of EXTs (Table 1). We used the 2,2-difenil-1-picrilhidrazilo radical (DPPH) technique, due to the stability of the DPPH free radical [27] and its scavenging capacity for radicals in extracts, as shown in [28]. QUE extract was used as an antioxidant activity control and showed a high reduction power for the DPPH radical (77.07 ± 0.74), expressed as inhibition %. BOL had an elevated antiradical power (82.83 ± 2.86). No difference was observed in antioxidant activity between both extracts.

We considered it important to determine the polyphenol and flavonoid contents in EXTs since their antioxidant capacity could be attributable to these compounds (Table 1). The QUE extract showed a high bioactive content.

### 2.2. Cellular Viability

**Figure 1:**
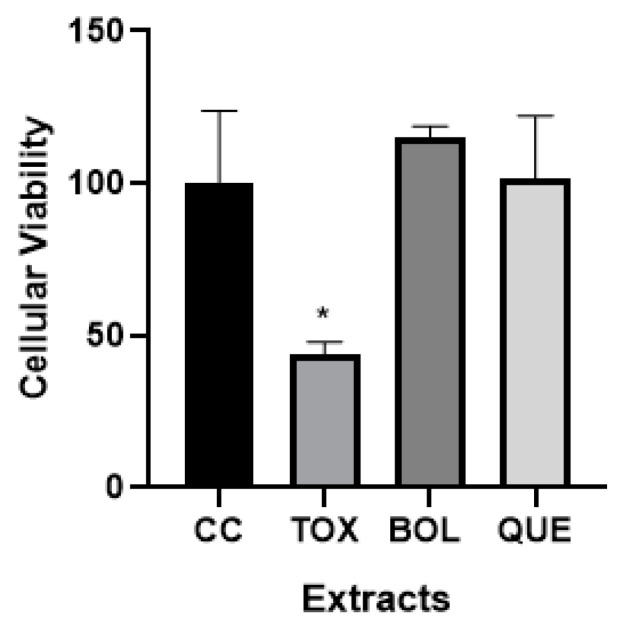
Effect of Boldo (BOL) and Quebracho (QUE) extracts (EXTs) on viability in 3T3-L1 preadipocytes. Cells were treated with doses of EXTs: 2 µg of GAE/mL (BOL) and 78 µg of GAE/mL (QUE). 3T3-L1 preadipocytes without differentiation treatment were considered control cells (CC), and toxicity control with H_2_O_2_ was performed (TOX). Absorbance of non-treated cells was set to 100; values are represented compared to them. Results are average of three different experiments (mean ± SD). * *p* < 0.05.

We observed that EXTs have a non-toxic effect (Figure 1) after 24 h of treatment, corresponding to the next polyphenol concentration expressed as 2 µg of GAE/mL (BOL) and 78 µg of GAE/mL (QUE). We decided to perform the EXT experiments in vitro at this dose. Only the toxicity control [TOX] showed a significant difference compared to the control cells [CC]. CC absorbance was set at 100 (100 ± 11 [CC] vs. 43 ± 2 [TOX], *p* < 0.05) and the values of BOL (115 ± 2) and QUE (101 ± 10) did not show a significant difference with respect to CC.

In fact, crude extract, which contains 0.20 mg of GAE/mL for Boldo and 7.82 mg of GAE/mL for Quebracho, showed an important in vitro cytotoxicity (63 ± 6 [Boldo] and 39 ± 7 [Quebracho] vs. 100 ± 11 [CC], *p* < 0.05). Therefore, the original samples of the extracts were diluted in order to use the non-toxic extracts for the cells.

### 2.3. In Vitro Antioxidant Effect in 3T3-L1 Cells

#### Intracellular ROS and Catalase Activity

**Figure 2:**
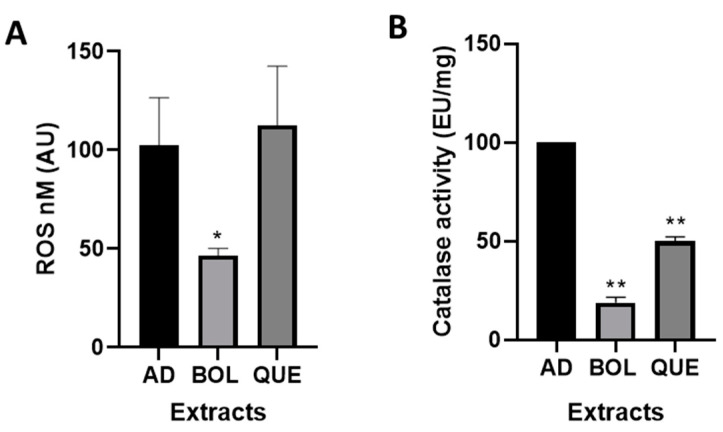
(**A**) Reactive oxygen species (ROS) intracellular content of adipocytes treated with Boldo (BOL) and Quebracho (QUE) extracts (EXTs). 3T3-L1 adipocytes were incubated for 24 h with EXTs. Cells were treated with doses of EXTs: 2 µg of GAE/mL (BOL) and 78 µg of GAE/mL (QUE). Results were expressed as R0S (nmol), compared to non-treated adipocytes (AD) considered as 100%. Each bar represents mean ± SD of triplicate experiment. * *p* < 0.05. (**B**) Catalase (CAT) activity of adipocytes treated with EXTs. 3T3-L1 adipocytes were incubated for 24 h with EXTs. Results were expressed as Enzymatic Units (EU/mg), compared to non-treated adipocytes (AD) considered as 100%. Each bar represents mean ± SD of triplicate experiment. ** *p* < 0.01 vs. AD.

We determined the ROS intracellular levels in AD and AD + EXTs after 24 h of treatment. We observed a significant decrease in ROS content in the BOL extract treatment compared with that for AD (46.50 ± 2.65 [AD + BOL] vs. 100 ± 17.20 [AD], *p* < 0.05). The QUE treatment did not show significant differences (Figure 2A), possibly because of the high content of tannins, which could fluoresce during the experiment.

We performed a catalase activity assay to evaluate the antioxidant power of EXTs in AD and AD + EXTs. BOL and QUE treatments, after 24 h, significantly decreased the activity of the catalase enzyme compared to AD, with *p* < 0.01 (Figure 2B) (18.81 ± 1.93 [AD + BOL], 49.95 ± 1.61 [AD + QUE], 100.00 ± 00.00 [AD]).

### 2.4. In Vitro Anti-Adipogenic Effect

#### 2.4.1. Neutral Lipid Content

**Figure 3:**
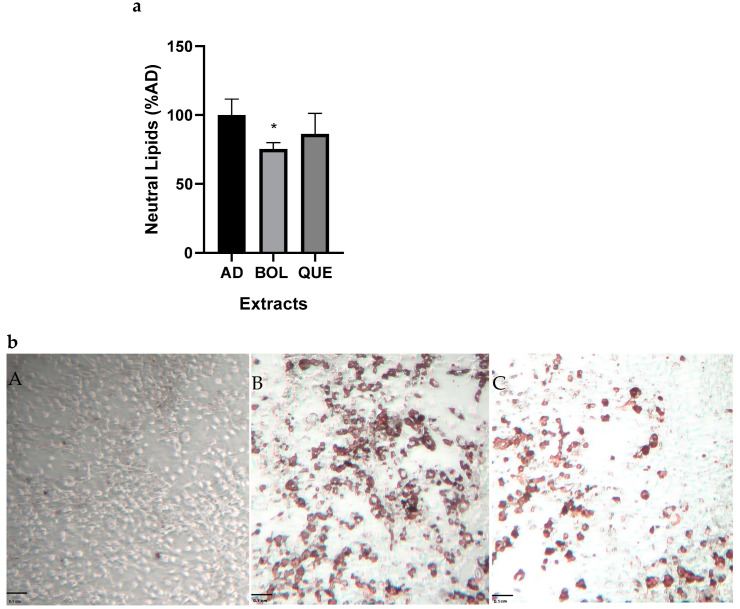
(**a**) Neutral lipid content of adipocytes treated with Boldo (BOL) and Quebracho (QUE) extracts (EXTs). Cells were incubated with EXTs for 24 h and stained with OIL RED O. Cells were treated with doses of EXTs: 2 µg of GAE/mL (BOL) and 78 µg of GAE/mL (QUE). Absorbance was measured at 590 ηm. Each bar represents mean ± SD of triplicate experiment. AD (non-treated adipocytes) is considered as 100%. * *p* < 0.05 vs. AD. (**b**) Oil Red O stained neutral lipid content of adipocytes treated with BOL extract. Cells were stained with ORO and photographed: (**A**) 3T3-L1 preadipocytes without differentiation treatment control cells; (**B**) 3T3-L1 non-treated adipocytes; (**C**) BOL extract 3T3-L1 treated adipocytes. Stained lipid droplets are shown (scale bar 0.1 cm). Representative results from one triplicate experiment with similar results are shown.

Based on the high antioxidant activities of some extracts, we decided to evaluate their effects on lipid accumulation in 3T3-L1 cells. We differentiated 3T3-L1 preadipocytes into mature adipocytes and evaluated the intracellular neutral lipid content via Oil Red O staining. We performed AD and AD + EXT treatments for 24 h. The EXTs were delivered by adding them to the culture media of adipocytes. BOL treatment significantly decreased the neutral lipid content compared to AD (Figure 3a,b) (100 ± 8.17 [AD] vs. 75.37 ± 3.33 [AD + BOL], *p* < 0.05).

#### 2.4.2. Intracellular Triglyceride Content

**Figure 4:**
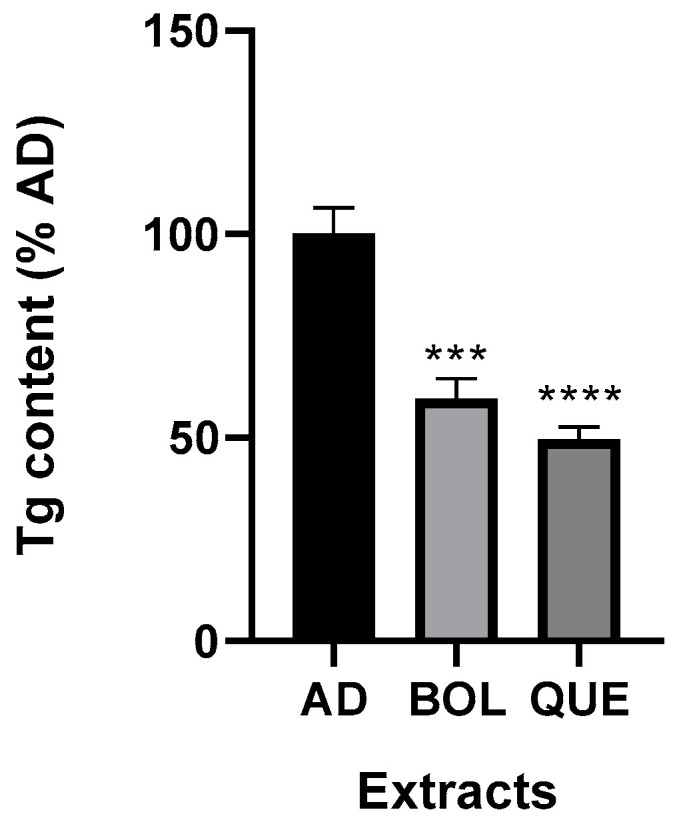
Triglyceride (Tg) content of adipocytes treated with Boldo (BOL) and Quebracho (QUE) extracts (EXTs). Intracellular Tg content was measured in AD (non-treated adipocytes) and AD + EXTs. Cells were incubated for 24 h with EXTs. Cells were treated with doses of EXTs: 2 µg of GAE/mL (BOL) and 78 µg of GAE/mL (QUE). Results were expressed as Tg content compared to AD (100%). Each bar represents mean ± SD of triplicate experiment. *** *p* ≤ 0.001; **** *p* ≤ 0.0001 vs. AD.

We performed AD and AD + EXT treatments for 24, 48, and 72 h to evaluate Tg intracellular accumulation. The EXTs were delivered by adding them to adipocyte culture media. BOL and QUE significantly (*p* ≤ 0.0001) reduced triglyceride levels after 24 h of treatment compared to AD non-treated samples. These results are shown as the percentage of non-treated adipocyte cells (AD) set at 100 (Figure 4). Tg content in AD non-treated samples was 2.01 ± 0.07 mg Tg/μg DNA; while Tg level in EXT-treated cells was significantly lower (1.19 ± 0.05 mg Tg/μg DNA [AD + BOL] and 0.99 ± 0.03 mg Tg/μg DNA [AD + QUE]). After 48 h of treatment with EXTs, only QUE decreased significantly in Tg content (*p* < 0.05); Tg content in AD was 41.66 ± 5.97 [AD + QUE] and 100.00 ± 4.95 [AD]. However, 72 h treatment with EXTs did not lead to significant differences.

#### 2.4.3. Intracellular Cholesterol Content

**Figure 5:**
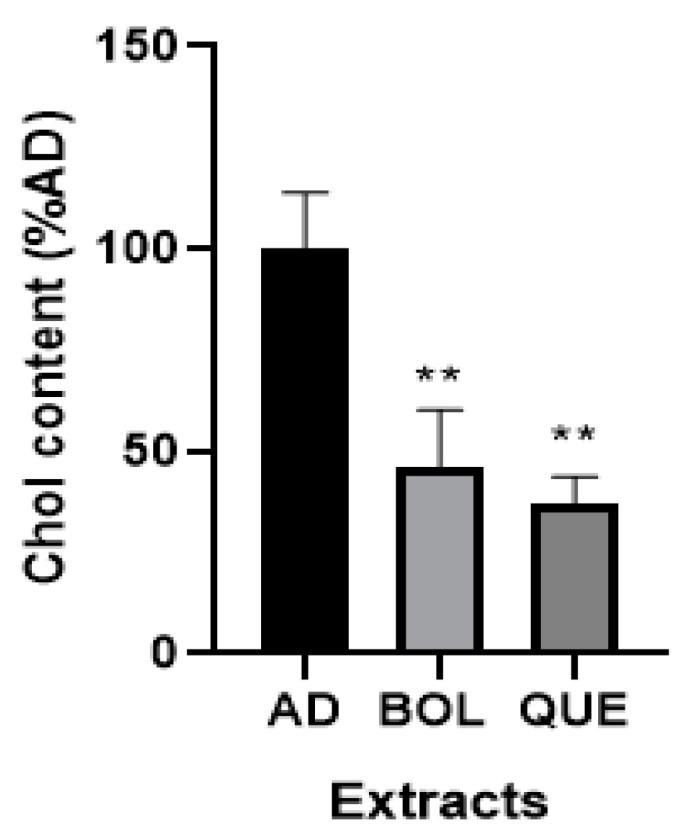
Cholesterol (Chol) content of 3T3-L1 adipocytes treated with Boldo (BOL) and Quebracho (QUE) extracts (EXTs). Intracellular Chol content was assessed in AD (non-treated adipocytes) and AD + EXTs for 24 h treatment with EXTs. Cells were treated with doses of EXTs: 2 µg of GAE/mL (BOL) and 78 µg of GAE/mL (QUE). Results were expressed as Chol content compared to AD (100%). Each bar represents mean ± SD of triplicate experiment. ** *p* < 0.01 vs. AD.

After 24 h treatment with EXTs in mature adipocytes, we evaluated Chol levels in the AD and AD + EXT samples. As shown in Figure 5, we observed that EXTs decreased intracellular Chol compared to non-treated AD (*p* < 0.01) (46.33 ± 7.98 [AD + BOL], 37.07 ± 3.79 [AD + QUE], 100.00 ± 7.97 [AD]). Intracellular Chol concentration in AD non-treated cells was 1.03 ± 0.08 mg Chol/μg DNA, while a significant decrease was observed for EXT-treated cells (0.47 ± 0.07 mg Chol/μg DNA [AD + BOL] and 0.39 ± 0.03 mg Chol/μg DNA [AD + QUE]).

#### 2.4.4. Glycerol Release

**Figure 6:**
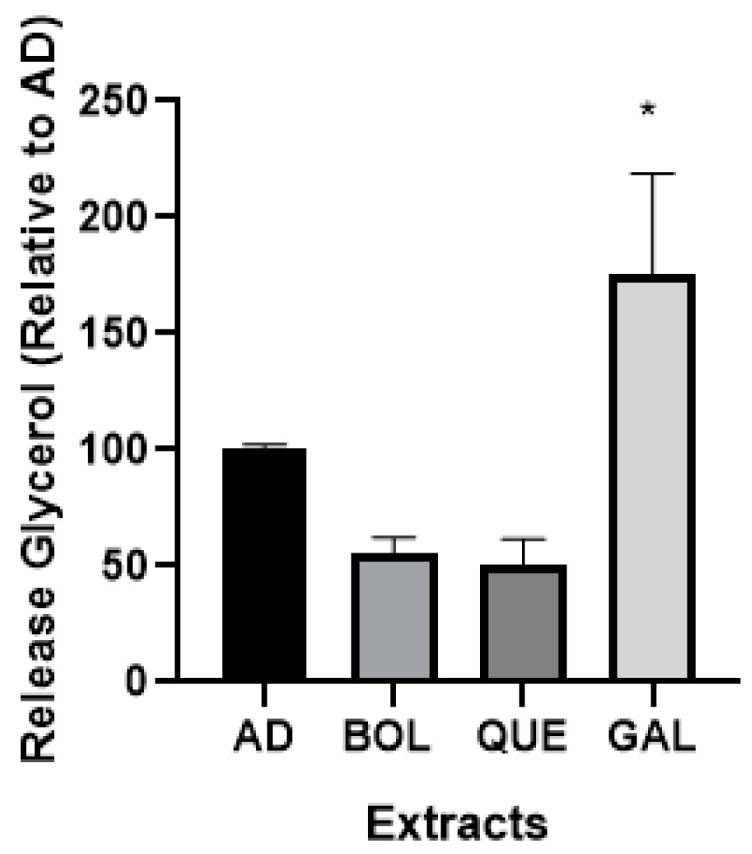
Glycerol (Gly) released from adipocytes treated with Boldo (BOL) and Quebracho (QUE) extracts (EXTs). Gallic acid (GAL) was used as bioactive control. Gly was assessed in culture medium from non-treated adipocytes (AD), AD + EXTs, and AD + GAL incubated for 24 h with EXTs. Cells were treated with doses of EXTs: 2 µg of GAE/mL (BOL) and 78 µg of GAE/mL (QUE). Results were expressed as % Gly relative to AD considered as 100%. Each bar represents mean ± SD of triplicate experiment. * *p* < 0.05 versus AD.

It has been reported that 3T3-L1 adipocytes release glycerol into the cell culture medium, a process referred to as basal lipolysis. We evaluated the possible effect of EXTs on lipolysis (Figure 6) after 24 h of treatment. Gallic acid (GAL) was used as a bioactive control. Only GAL treatment increased the levels of released glycerol (*p* < 0.05) (175.10 ± 30.97 [AD + GAL], 100.00 ± 1.60 [AD]).

#### 2.4.5. Nitrite Production

**Figure 7:**
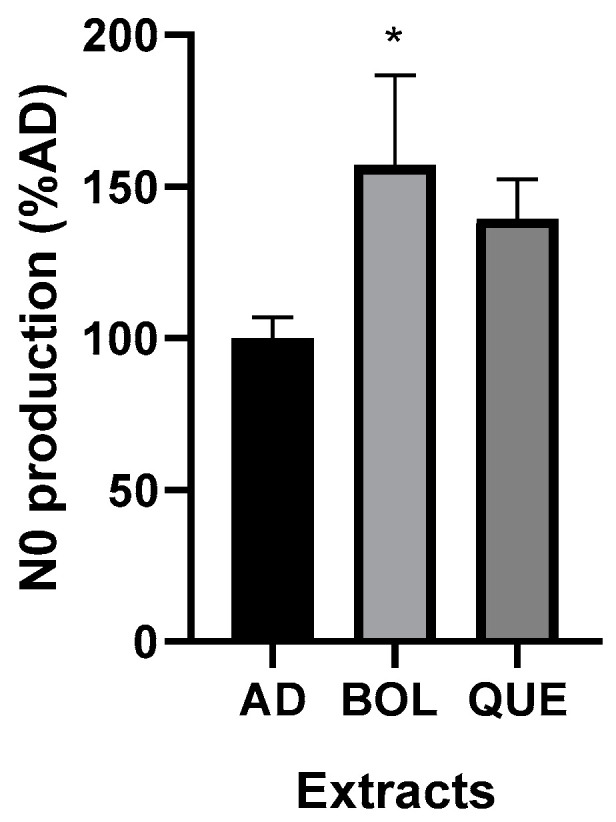
Endogenous nitric oxide (NO) produced by adipocytes treated with Boldo (BOL) and Quebracho (QUE) extracts (EXTs). NO was assessed in culture medium from non-treated adipocytes (AD) and AD + EXTs. 3T3-L1 adipocytes were incubated with EXTs for 24 h. Cells were treated with doses of EXTs: 2 µg of GAE/mL (BOL) and 78 µg of GAE/mL (QUE). Results were expressed as NO (μM), relative to AD considered as 100%. Each bar represents mean ± SD of triplicate experiment. * *p* < 0.05 versus AD.

We evaluated nitrite production in adipocytes 3T3-L1 after EXT treatment and its relationship with anti-adipogenic effects. We performed treatment with EXTs for 24 h. BOL extract significantly increased Nit production compared to non-treated AD (*p* < 0.05) (157.00 ± 21.02 [AD + BOL], 100.00 ± 3.98 [AD]), as well as QUE. The nitrite level in AD non-treated cells was significantly different to BOL treatment (15.17 ± 0.60 μM [AD] vs. 23.83 ± 2.12 μM [AD + BOL], *p* < 0.05). In the case of QUE, nitrite production was 21.17 ± 0.92 μM [AD + QUE]; no significant difference compared to AD was observed. These results are shown as the percentage of non-treated adipocyte cells (AD) set at 100 in Figure 7.

#### 2.4.6. TNF-α Secretion

**Figure 8:**
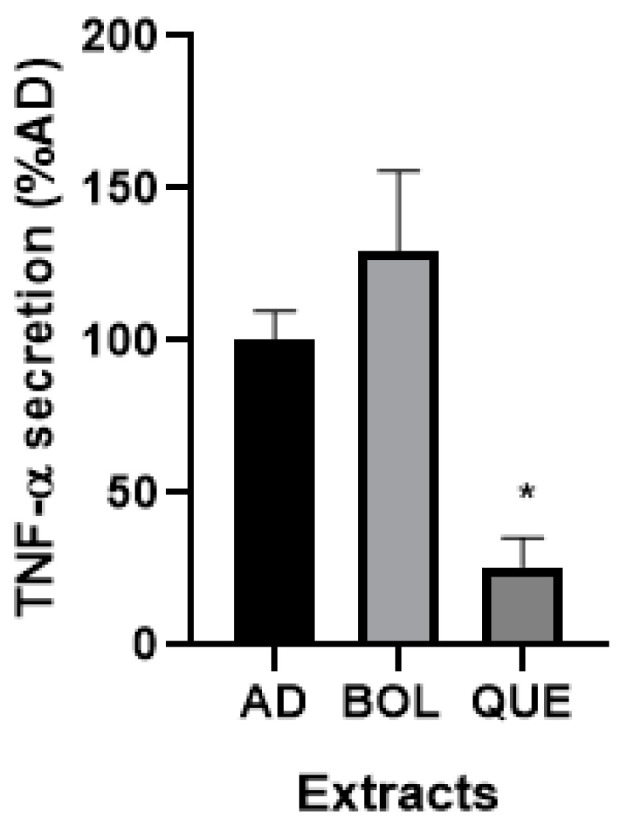
TNF-α secretion by adipocytes treated with Boldo (BOL) and Quebracho (QUE) extracts (EXTs). TNF-α levels were measured in culture medium of non-treated adipocytes (AD) and AD + EXTs treated for 24 h. Cells were treated with doses of EXTs: 2 µg of GAE/mL (BOL) and 78 µg of GAE/mL (QUE). Results were expressed as %TNF-α secretion (pg/mL) compared to AD (100%). Each bar represents mean ± SD of triplicate experiment. * *p* < 0.05 versus AD.

Since lipid accumulation is related to inflammation, we evaluated secreted TNF-α in our cells with high lipid content (AD) and AD + EXT (Figure 8). BOL extract did not significantly increase TNF-α secretion, while QUE extract decreased it, compared to non-treated AD (100.00 ± 8.53).

## 3. Discussion

Natural compounds may offer therapeutic potential for pathologies associated with oxidative stress, especially obesity [29]. Diets high in fruits and vegetables help to reduce the risk of obesity, metabolic syndrome, type 2 diabetes, cardiovascular disease (CVD), inflammation, among other diseases [30]. The mechanisms involve antioxidant and/or anti-inflammatory effects, such as scavenging free radicals and altering gene transcription through the activation or inhibition of transcription factors. Polyphenols (PPhs) are bioactive compounds present in vegetables; studies suggest that consuming PPhs may help to improve metabolic syndromes and contribute to the prevention of various chronic conditions, such as diabetes, obesity, hypertension, and colon cancer [31]. Moreover, an important comorbidity of obesity is dysfunction-associated steatotic liver disease (MALSD), which is considered the most common chronic liver pathology in the world, affecting more than 30% of the population [32]. In fact, there are no effective therapies for MASLD, but natural products appear as a possible alternative which could alleviate this pathology [33].

Boldo (*P. boldus*) is used in traditional medicine for its diverse biological effects, including its anti-inflammatory, hepatoprotective, and antioxidant properties [11]. We showed that the BOL extract presents a high antioxidant activity when using the DPPH radical inhibition technique. As a control for antioxidant activity, we used QUE extract, which showed similar activity. However, polyphenol and flavonoid contents in BOL were lower than those in QUE. Different methods can be utilized to evaluate antioxidant activity; of them, we used DPPH, a simple and quick technique. We analyzed the correlation between the antioxidant activities and Total Polyphenol Content (TPC) of various extracts of vegetable species in our region (*Baccharis articulata*, *Peumus boldus*, *Citrus sinensis*, *Schinopsis balansae*, and *Verbena bonaerensis*). The values showed a weak correlation (r^2^ = 0.35), similar to that previously observed by others between TPC and DPPH values (r^2^ = 0.58) [34].

Boldine is the main alkaloid in Boldo leaves [35] and has been detected in fractions of alcoholic extracts. Since we performed an aqueous extraction from Boldo leaves, boldine could not be detected in the samples 

Intending to evaluate the cellular effect on the oxidative metabolism of these extracts, we determined cellular ROS levels and catalase activity. During adipogenic differentiation, an increase in ROS levels occurred. Antioxidant molecules could inhibit ROS production through ROS scavenging [17]. Previously, we demonstrated that an antioxidant extract from Cassis (*Ribes nigrum*) could inhibit intracellular ROS accumulation. In this context, we suggested that the antioxidant properties of Cassis play a significant role in reducing oxidative stress [26]. In this study, we demonstrated a significant reduction in ROS levels in adipocytes treated with BOL extract compared to non-treated adipocytes.

In 3T3-L1 cells, hormone stimulation induces cell cycle progression, a necessary condition during adipogenesis; catalase (CAT) is an antioxidant enzyme that participates in the REDOX balance. CAT catalyzes the conversion of H_2_O_2_ into water and oxygen [36]. Since ROS levels are increased during adipogenic differentiation [37], CAT levels increase to avoid a possible apoptosis effect. Luteolin has been described as a dietary flavonoid with antioxidant and anti-adipogenic activities. Luteolin treatment decreased the CAT protein level during the differentiation process in 3T3-L1, suggesting that the antioxidant could be enough to prevent a cellular oxidative state without CAT involvement and, as a consequence, inhibit adipogenesis [38]. While Zhao et al. [38] used exogenous H_2_O_2_, we observed an endogenous increase in intracellular ROS in non-treated adipocytes and that BOL and QUE treatments produced a decrease in CAT activity. We suggest that BOL antioxidant activity may be responsible for the decrease in ROS levels and CAT activity in 3T3-L1 mature adipocytes. In the case of QUE, its effect on CAT would contribute to decreases in cellular Tg and Chol in adipocytes. It is important to consider that tannins in QUE may fluoresce; therefore, an increase in fluorescence could occur during the ROS experiment, which could result in an incorrect interpretation of the effect on ROS determination [39].

Obesity is a metabolic condition marked by an increase in lipids, primarily Tg and Chol. Antioxidants such as N-acetylcysteine (NAC) may reduce ROS production and lipid accumulation during the differentiation of 3T3-L1 preadipocytes [40] and mature adipocytes [4]. Since ROS increase during adipogenesis, the antioxidant environment created by NAC treatment is effective in inhibiting lipid accumulation. We employed the 3T3-L1 preadipocyte cell line, a model widely used for studying adipogenic differentiation [3]. We evaluated EXTs’ anti-adipogenic effects on mature adipocytes. We regarded the intracellular Tg content to be the primary endpoint of adipogenesis [41]; at this cellular stage, lipid droplets can be observed in mature adipocytes using an optical microscope. Oil Red O (ORO) staining is used to stain neutral lipids in cells as adipocytes and visualize lipid droplets [42]. Here, we observed a slight decrease in neutral lipid content in BOL-treated adipocytes using the ORO technique. The intracellular levels of Tg and Chol significantly decreased in EXT-treated adipocytes. Under these conditions, we suggest that the antioxidant properties of EXTs, through their bioactive components, are effective in reducing lipid accumulation in our model. This is the first report on the effects of the aqueous extract of Boldo on triglyceride and cholesterol content in adipocytes. The free radical activity of BOL could be primarily due to polyphenols (PPhs) such as catechins and flavonoids rather than boldine because of the relatively higher concentrations of these compounds in Boldo extracts [12]. Additionally, *P. boldus* extracts could serve as a natural potential antioxidant, particularly when leaf tissue is used in the extraction procedure [11]. We evaluated the potential lipolytic effects of BOL and QUE extracts in treated adipocytes by evaluating the release of glycerol into the culture medium. As shown in Figure 6, both extracts did not increase the released glycerol levels compared with AD (non-treated adipocytes), while the GAL control stimulated lipolysis, increasing the glycerol levels in the culture medium of treated 3T3-L1 adipocytes by almost 75%. As gallic acid (GAL) is a pure bioactive, we hypothesize that this condition supports the lipolytic effect. The extracts are a complex mixture of bioactives; it is possible that, for this reason, they did not show a lipolytic effect.

Finally, we evaluated some inflammation markers such as TNF-α and nitric oxide (NO). While the TNF-α level was significantly decreased by QUE, both EXTs increased NO levels. A possible explanation for this effect could be the different polyphenol contents for both EXTs. It is worth noting that, here, we studied the endogenous production of NO, in contrast with other authors who analyzed treatments with exogenous NO in 3T3-L1 adipocytes [19]. We performed treatments to evaluate endogenous nitric oxide (NO) in BOL- and QUE-treated adipocytes. This is not the first time that basal NO production has been reported [43]. However, this is the first report concerning NO and its anti-adipogenic effect, as other authors did not analyze basal production [19]. This kind of study could reveal the importance of this metabolic product related to lipid content in adipocytes. They showed that exogenous NO inhibits adipocyte differentiation in 3T3-L1 cells by blocking PPARγ function, according to ap2 and LPL decreases. Here, EXTs increased NO levels compared to AD, while BOL treatment showed significant differences. We suggest that the anti-adipogenic effect of BOL could also be related to increased NO levels.

## 4. Materials and Methods

### 4.1. Materials

Vegetable materials of *Peumus boldus* (Boldo) and *Schinopsis balansae* (Quebracho) were collected in Buenos Aires Province, Argentina (latitude 34°28′16″ S, longitude 58°45′29″ W) during the vegetative phase; they were identified as such by botanical faculty members. We obtained samples from 5 different plants for each vegetable material; the plant material was air-dried and ground to a size of less than 500 µm. With the aim of obtaining homogenous material, samples of Boldo were mixed together to obtain a composite mixture of plant material, and the same was carried out for the Quebracho samples. The obtained powder was used immediately for the extraction process.

The murine 3T3-L1 preadipocyte cell line was obtained from American Type Culture Collection (Rockville, MD, USA). Gallic acid and rutin were purchased from Sigma Aldrich Inc., St. Louis, MO, USA.

### 4.2. Extract Preparation

Boldo (BOL) and Quebracho (QUE) plant materials were stored in the dark at room temperature until use. The phosphate-buffered saline (PBS) aqueous extracts (EXTs) were obtained from leaves of BOL and QUE cortex for 1 h at 37 °C; 0.01 g of each composite mixture in 1 mL of PBS were used for the incubations. Next, they were each centrifuged at 10,000 RPM for 15 min. The liquid phase was separated and conserved at 4 °C for 72 h. Then, the EXTs were also filtered through a 0.22 μm membrane and frozen at −18 °C until use.

### 4.3. EXT Characterization

The EXTs were physically and chemically characterized as follows: 1—The acidity was determined with a digital pH meter (pH 211, HANNA Instrument, Nusfalau, Romania). 2—The antioxidant capacity was determined via a previously described method [44] using the 2,2-diphenyl-1-picrilhidrazilo (DPPH) technique [27], and the results were expressed as a percentage of DPPH free radical scavenging; QUE was used as an antioxidant control. 3—The total phenol content was quantified via the previously described Folin colorimetric technique [45] using gallic acid (2 mg/mL) as the standard. 4—The total flavonoid concentration was determined via the aluminum chloride procedure, using rutin (1 mg/mL) as the standard solution [46]. 5—The glucose concentration in the sample was evaluated via the glucose-oxidase method. The working reagent was GOD-POD (Liquid AA Enzymatic Glycemia kit—Wiener Laboratory SRL, Rosario, Argentina).

### 4.4. Cell Culture and Differentiation of 3T3-L1 Preadipocytes

3T3-L1 cells were cultured in DMEM supplemented with 10% fetal bovine serum (FBS), vitamins (Sigma Aldrich, Inc., St. Louis, MO, USA), non-essential amino acid (Sigma Aldrich Inc., St. Louis, MO, USA), and antibiotics (penicillin 1%, streptomycin 1%, Sigma Aldrich Inc., St. Louis, MO, USA). This medium is called complete DMEM (DMEMc). The culture conditions were set at 37 °C in a 5% CO_2_ incubator. Adipogenesis in 3T3-L1 cells was induced with a differentiation medium containing 0.5 mM 3-isobutyl-1-methylxanthine, 0.1 μM dexamethasone, and 2 μM insulin into DMEMc for 72 h. Then, the cells were transferred to fresh DMEMc containing two μM insulin for 72 h. Next, the cells were maintained in DMEMc until the adipocytes had fully developed (mature adipocytes). Then, after 10 days of differentiation, we observed intracellular lipid droplets in an optical microscope at a total magnification of 400× [47].

### 4.5. In Vitro Treatment

We treated mature adipocytes (AD) with the EXTs to evaluate the different parameters. 3T3-L1 preadipocytes without differentiation treatment were considered the control cells (CC).

### 4.6. Cytotoxicity Evaluation

To determine cell viability, we used the MTT technique. To this end, we evaluated the toxicity effect of EXT treatment in 3T3-L1 preadipocytes. This assay consisted of an oxide-reduction reaction in which mitochondrial enzymes of viable cells reduced MTT, resulting in a purple-colored product [48]. We seeded 45,000 cells/well in plates with 24 wells, cultured in DMEMc during the experiments. After reaction, the resulting solution in each well was transferred to a 96-well plate, and its absorbance was measured at 550 nm. We evaluated the EXTs using the original extracts (0.20 mg GAE/mL and 7.82 mg GAE/mL for BOL and QUE, respectively) and different dilutions of the original extracts in DMEMc: 20 µg and 2 μg GAE/mL (BOL) and 780 µg and 78 µg GAE/mL (QUE). We used 50 mM of H_2_O_2_ as a toxicity control and DMEMc as a viability control, as previously reported [49].

### 4.7. Determination of Intracellular ROS

The ROS assay was performed as previously described [50]. We evaluated CC, AD, and AD + EXT after treatment. The cells were separated from the multiwell plate using trypsin, washed with PBS, and used immediately in a fluorescence assay with a CM-H2DCFDA cell-permeable probe (Sigma Aldrich, Inc., USA). This probe emits fluorescence under oxidation (λ excitation = 505 nm, λ emission = 520 nm). The results of the treatments were normalized by DNA content and expressed relative to non-treated AD set as AU 100%.

### 4.8. Determination of Catalase Activity

Catalase activity assay was performed as previously described [51]. The cells were detached from the culture dish via scrapping. The reagents used were phosphate buffer (PB) (50 mM, pH 7) and hydrogen peroxide (H_2_O_2_) 30 mM. We mixed 50 μL of each sample and the kinetics were measured at 240 nm via spectrophotometry. The results of the treatments were normalized by DNA content and expressed relative to non-treated AD set at AU 100%.

### 4.9. Oil Red O Staining

Oil Red O (ORO) staining was realized as described previously [52]. We evaluated CC, AD, and AD + EXT after treatment. The cells were washed three times with PBS and fixed with 4% formaldehyde for 20 min at room temperature (RT). After another wash, we prepared an ORO work solution (0.4%), which was filtered using a 0.22 μm membrane. We added 300 μL of the work solution and incubated it in the dark for 2 h at RT in constant agitation. Next, the cells were washed thoroughly, and the stained lipid droplets in the cells were visualized via an optical microscope and then photographed. Afterward, the stained cells were extracted with isopropanol, and their absorbance was determined at 510 nm to quantify the staining. The results were expressed relative to non-treated AD set as AU 100%.

### 4.10. Determination of Triglycerides and Cholesterol

Tg and Chol accumulation was measured using a TG color GPO/PAP AA kit (Wiener Laboratory, Rosario, Argentina) and a Colestat AA kit (Wiener Laboratory, Rosario, Argentina), respectively. We evaluated CC, AD, and AD + EXT after treatment. The cells were lysed via hypotonic shock and via scrapping to obtain the samples and evaluate these lipids. Tg and Chol levels were normalized by DNA content. The results were expressed relative to non-treated AD set as AU 100%.

### 4.11. Determination of Glycerol Released to Culture Medium

We evaluated CC, AD, and AD + EXT after treatment. We conserved the cell culture medium to measure the released glycerol. We performed three extractions with a mixture of chloroform/methanol (2:1) to remove the triglycerides present in the medium, and glycerol content was assessed using a commercial kit from Wiener Laboratory [49]. The results were normalized with DNA content. They were expressed relative to non-treated AD and set as AU 100%.

### 4.12. Nitrite Determination

We evaluated CC, AD, and AD + EXT after treatment. We conserved the cell culture medium to measure nitrites via Griess reagent [53]. The results were normalized to the DNA content and were expressed relative to non-treated AD, set as AU 100%.

### 4.13. TNF-α Determination

To evaluate TNF-α secretion, we conserved (−80 °C) CC, AD, and AD + EXT after treatment. We used a Commercial Kit Mouse TNF alpha Uncoated ELISA (Invitrogen, Thermo Fisher, Wien, Austria).

### 4.14. Statistical Analysis

All the results are expressed as means ± standard deviation (SD) from three or four independent experiments. Statistical analyses were performed using one-way analysis of variance, followed by Dunnett’s post hoc analysis.

## 5. Conclusions and Future Directions

It is not possible to extrapolate any conclusion from the results obtained in this in vitro study to an in vivo situation. Nevertheless, no toxic effect has been reported in humans consuming Boldo teas using 3 g of dry leaves (tea bags) in 250 mL of water. In this study, we evaluated a lower dose. Besides that, toxicological clinical trials are needed to evaluate the possible toxic effects of Boldo. In this regard, recently, a case report was published explaining that Boldo leaves tea could induce hepatotoxicity [54].

It is important to mention that only a few interactions between Boldo and different kinds of medications were reported. But none of them are related to drugs used in the treatment of obesity or hypercholesterolemia. Boldo could increase warfarin anticoagulant effect [55] and diminish the tacrolimus immunosuppressive levels used in surgeries [56].

Our results suggested that *Peumus boldus* has important medicinal potential, with significant benefits for decreasing lipid accumulation in adipocytes. However, understanding the molecular mechanism in adipocyte differentiation is crucial, and it is still an open study to perform, mainly related to its potential use in the development of innovative therapies.

## Figures and Tables

**Table 1:** Antioxidant activity and polyphenol content of BOL and QUE crude extracts.

Extracts	Antioxidant Activity	Polyphenols (mg GAE/mL)	Flavonoids (mg RE/mL)
Boldo	82.83 ± 2.86	0.20 ± 0.00	1.30 ± 0.02
Quebracho	77.07 ± 0.74	7.82 ± 0.17	5.32 ± 0.18

Characteristics of extracts Boldo (BOL) and Quebracho (QUE). Original samples of extracts were evaluated: antioxidant activity was expressed as percentage of DPPH free radical scavenging and total contents of compounds were determined (MEAN ± SD of triplicate experiment). GAE: gallic acid; RE: rutin; SD: standard deviation.

## Data Availability

The data obtained and analyzed in this manuscript are available from the corresponding author upon request.
